# Neo: an object model for handling electrophysiology data in multiple formats

**DOI:** 10.3389/fninf.2014.00010

**Published:** 2014-02-20

**Authors:** Samuel Garcia, Domenico Guarino, Florent Jaillet, Todd Jennings, Robert Pröpper, Philipp L. Rautenberg, Chris C. Rodgers, Andrey Sobolev, Thomas Wachtler, Pierre Yger, Andrew P. Davison

**Affiliations:** ^1^Centre de Recherche en Neuroscience de Lyon, CNRS UMR5292–INSERM U1028–Université Claude Bernard Lyon 1Lyon, France; ^2^Unité de Neurosciences, Information et Complexité, Neuroinformatics group, CNRS UPR 3293Gif-sur-Yvette, France; ^3^Institut de Neurosciences de la Timone UMR 7289, Aix Marseille Université, CNRSMarseille, France; ^4^Division of Neurobiology, Department Biology II, Ludwig-Maximilians-Universität MünchenPlanegg-Martinsried, Germany; ^5^Neural Information Processing Group, TU BerlinBerlin, Germany; ^6^G-Node, Department Biology II, Ludwig-Maximilians-Universität MünchenPlanegg-Martinsried, Germany; ^7^Helen Wills Neuroscience Institute, University of CaliforniaBerkeley, CA, USA

**Keywords:** electrophysiology, interoperability, Python, software, file formats

## Abstract

Neuroscientists use many different software tools to acquire, analyze and visualize electrophysiological signals. However, incompatible data models and file formats make it difficult to exchange data between these tools. This reduces scientific productivity, renders potentially useful analysis methods inaccessible and impedes collaboration between labs. A common representation of the core data would improve interoperability and facilitate data-sharing. To that end, we propose here a language-independent object model, named “Neo,” suitable for representing data acquired from electroencephalographic, intracellular, or extracellular recordings, or generated from simulations. As a concrete instantiation of this object model we have developed an open source implementation in the Python programming language. In addition to representing electrophysiology data in memory for the purposes of analysis and visualization, the Python implementation provides a set of input/output (IO) modules for reading/writing the data from/to a variety of commonly used file formats. Support is included for formats produced by most of the major manufacturers of electrophysiology recording equipment and also for more generic formats such as MATLAB. Data representation and data analysis are conceptually separate: it is easier to write robust analysis code if it is focused on analysis and relies on an underlying package to handle data representation. For that reason, and also to be as lightweight as possible, the Neo object model and the associated Python package are deliberately limited to representation of data, with no functions for data analysis or visualization. Software for neurophysiology data analysis and visualization built on top of Neo automatically gains the benefits of interoperability, easier data sharing and automatic format conversion; there is already a burgeoning ecosystem of such tools. We intend that Neo should become the standard basis for Python tools in neurophysiology.

## 1. Introduction

Neuroscience research relies on specialized software to analyze and visualize data, but often no single piece of software is sufficient to meet all the needs of a particular study. The solution is either to extend the software or to transfer data between software tools. The former requires both programming skills and effort. If an entirely novel analysis method is needed, this effort is unavoidable, but if, as is often the case, the required functionality is already provided by a different software tool, transferring the data can in principle be much more efficient. Unfortunately, in neurophysiology there is a very wide variety of software in use, much of it proprietary and developed by recording hardware manufacturers, and with little in the way of common data formats. In practice, researchers spend a lot of time and effort converting data between different formats, and those with good programming skills are strongly tempted to re-implement functionality that already exists elsewhere rather than go through a tedious conversion process. A reduction in the amount of effort required for neurophysiology data conversion could greatly enhance the productivity of the field and reduce duplication of effort.

Three approaches to reducing the effort of data conversion, and thereby increasing the interoperability of data analysis tools, can be envisioned. The first is to develop a common file format, capable of containing all data generated by all the different software tools in use. Having such a file format, the number of possible conversion pathways that must be implemented is greatly reduced. Ideally, hardware manufacturers would support exporting recorded data in this format. This approach has been tried with some success in the domain of clinical electroencephalography (EEG) (Kemp et al., 1992; Kemp and Olivan, 2003; Schlögl, 2006), and the Program on Standards for Data Sharing of the International Neuroinformatics Coordinating Facility (INCF) is currently developing a standard file format for electrophysiology data (Teeters et al., 2013) based on the HDF5 file format. A comparison of 19 different biomedical data formats is provided by Schlögl (2010).

The second approach is to define a language that can describe the structure of data files in different formats, and then write descriptions of each of the formats of interest using this language. Conversion between data formats, or reading a data file into software, can then be fully automated. This approach was taken by Durka and Ircha (2004), who developed the SignalML markup language for describing biomedical time series.

The third approach is to define an application programming interface (API) that takes care of the data conversion, and implement this as a library which can be used by software developers to provide uniform access to data, independent of the file format. An advantage of this compared to the other two approaches mentioned above is that it encourages modularity in software development: the data-conversion library defines certain data structures which can then be used by developers of analysis and visualization tools, which thereby gain greater interoperability. A previous effort to define an API for electrophysiology data in neuroscience was undertaken by a consortium of recording hardware manufacturers, which in 2000 began development of the Neuroshare API (http://neuroshare.org). The Neuroshare API is available as a C library and as a MATLAB (The Mathworks Inc.) extension. These are distributed as compiled dynamic-link libraries (DLLs), one per manufacturer; the source code is generally not available, and most are only available for the Windows operating system. The API is rather low-level, consisting of a number of functions and low-level data structures such as numerical arrays. BioSig (http://biosig.sourceforge.net/) is a cross-platform open source library for biomedical signal processing (EEG, ECG, etc.), available for MATLAB/Octave and for C/C++ (with wrappers for Python), which includes support for a large number of biomedical file formats.

We have developed a novel, object-oriented API for representing electrophysiology data in memory and for reading/writing such data from/to multiple file formats. The Neo (from Neuroscience Electrophysiology Objects) API is defined as an object model, and is intended to be able to represent any electrophysiology dataset, from *in vitro* patch-clamp recordings through multi-electrode array recordings to EEG. We have implemented the Neo object model as a cross-platform, open-source Python package, but it should be possible to implement it in any object-oriented programming language. An object-oriented API is a natural fit for neuroscience data, and in particular makes it easier to encapsulate data and the associated metadata, hence preserving the information about the experimental context that is needed to correctly interpret and analyze the data.

The principal goal of developing Neo is to improve the interoperability of software for working with electrophysiology data. Such interoperability includes the exchange of data objects between software tools in memory as well as via the filesystem. The existence of a Neo implementation in a given language means, for example, that one scientist or research group can develop a library for electrophysiology data analysis and another a library for visualization of such data, then a third scientist can easily combine both libraries in his/her own workflow, without having to worry about plumbing the tools together, taking care of correct metadata conversion, etc. By having an object model that is shared across programming languages, we hope to make conversion of data analysis routines between languages more straightforward.

In this article we describe the considerations which led to the design of the API, we describe the object model and its Python implementation, then give some examples of using the Neo Python package in real-world situations. Finally, we discuss the advantages and disadvantages of the Neo approach with respect to the other approaches to data sharing and software interoperability mentioned above, and outline possible future directions.

## 2. Design considerations

Starting with the basic requirement that the object model should allow the representation and manipulation of any electrophysiology dataset, we elaborated the design according to the following principles.

### 2.1. Scope

It was decided that the object model should contain only classes for representing electrophysiology data and for reading/writing such data from/to files or databases. Further, the behavior of these objects should allow only basic manipulations such as rearranging datasets or extracting sub-sets. In particular, we wished to specifically exclude visualization or analysis methods, even such simple ones as taking the mean. The motivation for this was to keep Neo as lightweight as possible. In our experience developing open-source software, small, focused libraries are much more likely to see widespread uptake. In future, we expect to extend the object model to support other data types closely-related to electrophysiology, such as calcium imaging and behavioral data, but these were not included in the initial design so as to avoid diluting our efforts.

### 2.2. Metadata

We distinguish *essential* metadata, which is required for the data to have even minimal meaning rather than just be arrays of numbers, *desirable* metadata, which is necessary to correctly analyze the data, and *additional* metadata. Examples of essential metadata are the sampling interval and the physical units of a recording. Desirable metadata might include identification of the neuron or brain area from which the recording was made, or the filter settings used. Additional metadata provide information that is specific to a particular user or software tool, such as which color to use when plotting the data in a graphical interface. The object model should make it impossible to create data objects without essential metadata. For desirable and additional metadata, it should be straightforward to annotate any object. Annotations should take the form either of key–value pairs or of subject–predicate–object triples as used in RDF (Manola and Miller, 2004). In our Python implementation, we chose key–value pairs for simplicity, but we are considering adding support for triples in future versions. For desirable metadata we add the further requirement that the metadata must be preserved when writing to and then reading again from a file or database, whatever the format.

### 2.3. Structure

Experimental protocols in neuroscience are hugely diverse, and may be structured in many ways in terms of stimulus presentation, behavioral responses, and pharmacological interventions. The object model should allow at least the structure related to the electrophysiological recordings to be represented in the relationships between data objects rather than only as metadata. At least two levels of hierarchical organization are needed (e.g., a recording session consisting of multiple trials, or a multi-electrode recording with multiple channels), but non-hierarchical cross-links are also needed.

### 2.4. Interoperability

Wherever possible, compatibility with other widely used scientific libraries in a given implementation language should be preserved.

### 2.5. Efficiency

Electrophysiology datasets are often very large, either because recording lasts for a long time, hours or days, or because of recording from many channels. This means that both the object model and its implementation must provide memory- and computation-efficient representations.

## 3. Object model

**Figure 1:**
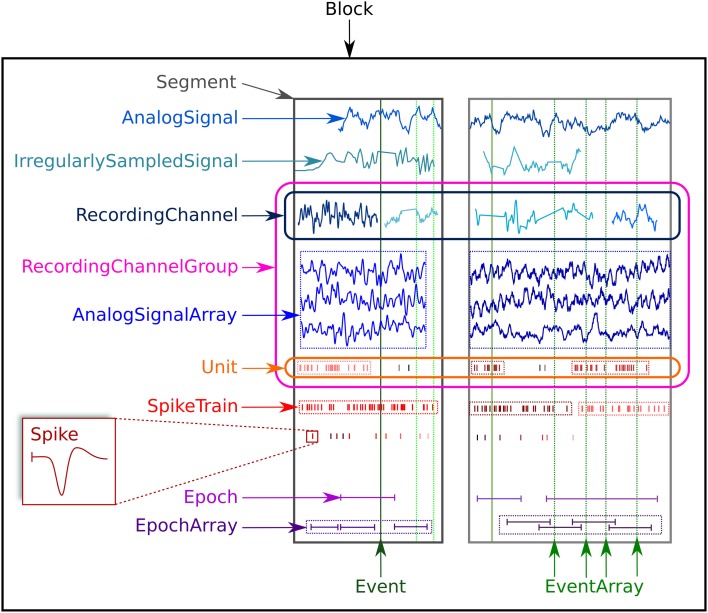
**An illustration of the data types supported by Neo and their grouping into containers**.

The Neo object model consists of 14 classes, which may be divided into three types: data objects, container objects and grouping objects. Figure 1 provides an overview of the Neo objects and their relationships. Data objects contain numerical data together with associated metadata. The data types that are represented are (i) sampled continuous, analog signals; (ii) action potentials (“spikes”), characterized by their time of occurrence and, optionally, their waveforms; (iii) events, each of which consists of a time of occurrence and a textual label; and (iv) epochs, each of which consists of a start and end time together with a label. The full list of data objects is as follows:

- AnalogSignal: a record of a continuous, analog signal with a fixed sampling interval, for example the membrane potential recorded from an intracellular electrode using current clamp.
- IrregularlySampledSignal: a record of a continuous analog signal with a varying sampling interval. This is found, for example, in data from neuronal network simulations with a variable-time-step integration method.
- AnalogSignalArray: a record of a multichannel continuous analog signal with a fixed sampling interval, for example from an multi-electrode array or from an EEG recording.
- Spike: one action potential characterized by its time and waveform.
- SpikeTrain: a set of action potentials (spikes) emitted by the same unit in a period of time (with optional waveforms). Such data is generated by spike sorting from multi-electrode recordings, and directly by simulations.
- Event and EventArray: a time point representing an event in the data, or an array of such time points. Events may represent, for example, pharmacological interventions or triggers during behavioral experiments.
- Epoch and EpochArray: an interval of time representing a period of time in the data, or an array of such intervals. An example would be the time during which a stimulus is presented.

For each data type, the object model provides both a scalar representation (a single instance of a data item, for example, AnalogSignal and Spike) and an array representation (e.g., AnalogSignalArray and SpikeTrain) in order to provide a choice between simplicity and efficiency in data analysis. For example, when working with single-electrode data, using the scalar objects is simpler and produces code that is easier to understand, without having to deal with array indexing. For electrode-array or EEG data on the other hand, using NumPy-like arrays is much more efficient than using lists of objects.

Container objects provide a simple hierarchical grouping of data. A Segment may contain any of the data object types provided all the objects share a common clock, i.e., they represent data recorded at the same time, although the sampling interval, onset and duration of recording may differ between data objects. A Block is the top-level container, and contains Segments and RecordingChannelGroups (see below). To illustrate this further, in the common experimental paradigm of running multiple trials, i.e., presenting the same stimulus multiple times, the data from each trial would be stored in a Segment, and all the Segments stored in a single Block. Deeper or more complex hierarchies are not supported directly by Neo, but it would be straightforward for a user to define a higher-level object to contain multiple blocks. Some of the Neo input/output modules (see below) support storage of multiple Block objects.

Grouping objects express the relationships between data objects, such as which signals were recorded on which electrodes, which spike trains were obtained from which membrane potential signals, etc. They contain references to data objects that cut across the simple container hierarchy. Another way to think of this organization is that “container” objects group data that were acquired at the same time, while “grouping” objects group data that were recorded from the same channel, array, location, etc. The grouping objects are as follows:

- Unit: gathers all the Spike and SpikeTrain objects within a Block, possibly across several Segments, that were emitted by the same neuron.
- RecordingChannel: links AnalogSignal and IrregularlySampledSignal objects that come from the same logical and/or physical channel inside a Block, possibly across several Segment objects.
- RecordingChannelGroup: a group for associated RecordingChannel objects, for example all the channels from a single tetrode.

The RecordingChannelGroup has several uses. Firstly, for linking several AnalogSignalArray objects across several Segment objects, e.g., linking multi-electrode array recordings across trials. Secondly, to associate a Unit with the group of recording channels from which it was calculated (since with multi-electrode arrays, action potentials from the same neuron may be recorded on more than one recording channel); Thirdly, for grouping RecordingChannel objects by signal type (for example, with intracellular recording, it is common to record both membrane potentials and currents at the same time). Fourthly, for multi-electrode arrays, a RecordingChannelGroup is used to gather all RecordingChannel objects belonging to the same array.

### 3.1. Object attributes

Most objects, and in particular all of the data objects, have a number of *required* attributes. These are essential metadata without which the numerical data values contained in the object cannot be interpreted. For example, in the case of AnalogSignal, the required attributes are the sampling interval (or, equivalently, the sampling rate), the time stamp of the first sample and the units of the signal, for example “millivolts” in the case of the membrane potential. All attributes that represent physical quantities must state the units, for example, the sampling rate could be given as “10 kHz.”

In addition to the required attributes, each object has a number of *suggested* attributes. These attributes are intended to contain desirable metadata that are not essential for working with the objects, but that are likely to be useful in developing analysis methods, automatic graph generation, tracking provenance, etc. Examples of such suggested attributes are name, rec_datetime (the date and time of the original recording) and file_origin (the filesystem path or URL of the original data file from which the data were read). The number of suggested attributes is likely to increase in future versions as structured terminologies and ontologies for neurophysiology are developed. Full details about required and suggested attributes are available in the Neo documentation, online at http://neuralensemble.org/neo/.

Finally, any object may contain any number of additional attributes or annotations. Such annotations may be numerical or text values, arrays of such values, or dictionaries/hash tables containing such values. They are intended to contain any information that may be useful in subsequent processing of the data, for example metadata describing the experimental protocol.

### 3.2. Relationships between objects

**Figure 2:**
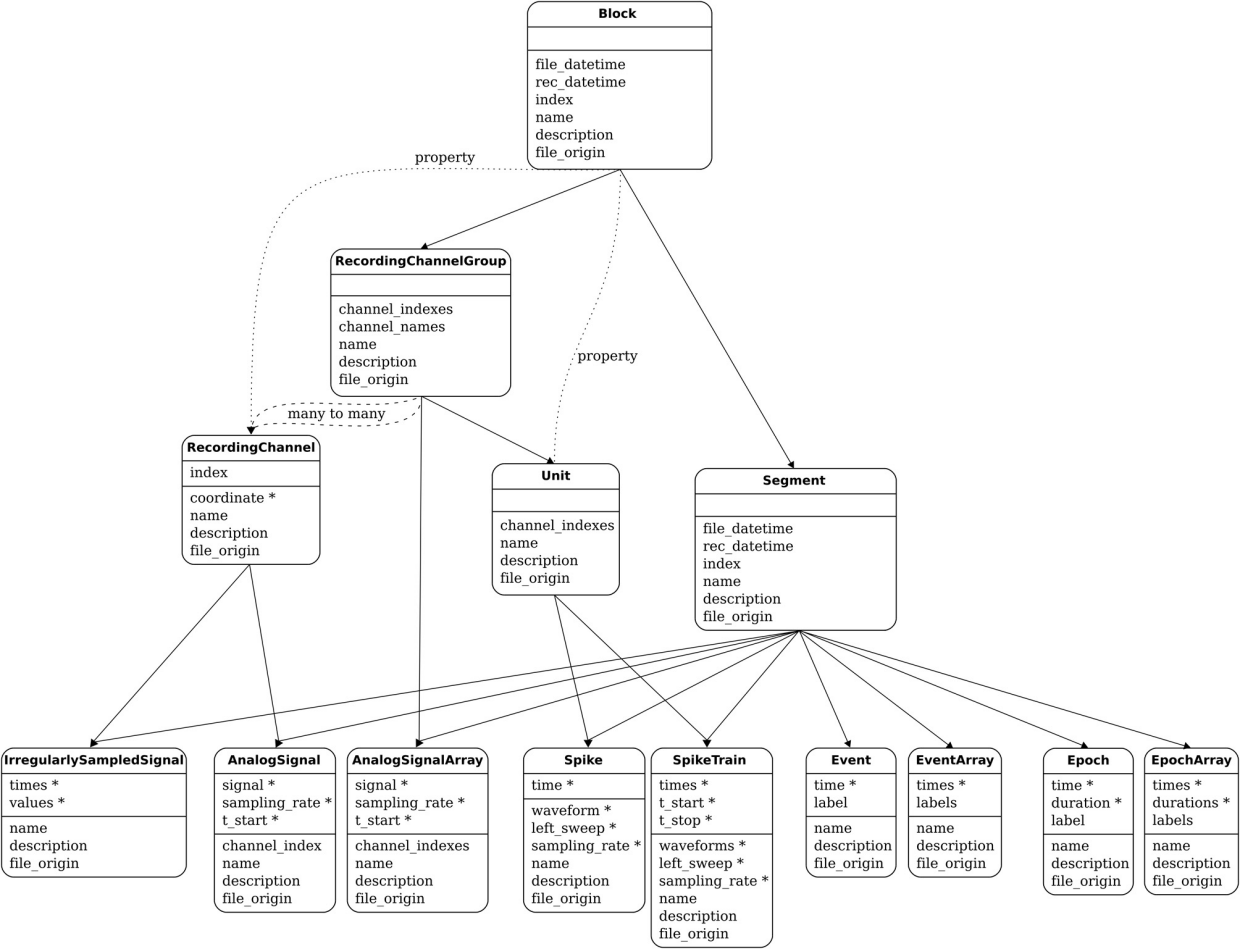
**The Neo object model: the principal classes and their relationships**. For each class, a horizontal line separates the required attributes from the suggested attributes. The stars mark the attributes that have an associated unit.

The functions of the container and grouping objects are based on lists of references to other objects. For example, Block has an attribute segments, which is a list of references to all the Segment objects contained in that Block. Such relationships are bi-directional, for example each Segment has an attribute block pointing to the Block within which it is contained. Figure 2 shows all of these relationships. More detail is available in the online documentation.

### 3.3. Implementation in python

When implementing the object model in a specific language, ease-of-use considerations come to the fore. If a package or library is to gain widespread adoption, it should be easy to build and install on different platforms, and should be interoperable, as much as possible, with existing tools and existing code for handling electrophysiology data in that language.

In the case of Python, the latter consideration dictates that a Neo implementation must be based on NumPy (Oliphant, 2007), the *de facto* standard for numerical computing in Python. NumPy provides a powerful, *N*-dimensional array object that can contain integers, floating point numbers, strings or other Python objects. A given numerical array can have any combination of precision and byte-order. Most array operations are implemented in C or Fortran for efficiency. The principal choice in implementing the Neo object model in Python is whether the classes for data objects should inherit from the NumPy ndarray class (a Neo class *is* a NumPy array) or whether to use composition (where the Neo class has a data attribute which is a NumPy array). Inheritance has two important advantages: (i) existing code that works with NumPy arrays will work with Neo data objects without modification; (ii) Neo objects gain most of the functionality of NumPy arrays “for free,” i.e., we do not have to re-implement this functionality. A number of operations on, and methods of, NumPy arrays have to be re-implemented or extended for Neo objects, in order to preserve the extra metadata carried by Neo objects, but these would in any case have to be implemented if taking the composition approach. The principal disadvantage of the inheritance approach is complexity: creation of subclasses is one of the more difficult parts of the NumPy API. After weighing up these considerations, we chose to use inheritance.

The second major choice was how to represent the measurement units of the data. Several Python packages are available for handling units and physical quantities in Python. The decision to use inheritance for NumPy support narrows down the choice. We chose to use the Quantities package (http://pythonhosted.org/quantities/), which provides a Quantity class that inherits from the NumPy ndarray class and adds a concept of physical dimensions and units. This means that trying to add a Quantity array with dimensions of voltage to an array with dimensions of current will produce an exception, while adding an array in millivolts to an array in volts will perform scaling so as to give the correct result. The particular choice of Quantities is not limiting. It would be possible to replace it with another units package that subclasses ndarray, such as Pint (http://pint.readthedocs.org/), without changing the Neo interface.

In summary, the classes for the Neo data objects all inherit from Quantity, and so gain checks for dimensional consistency for free, in addition to the large amount of functionality inherited from ndarray. Neo objects add further checks (for example, trying to add together two AnalogSignals with different sampling rates produces an exception) and further operations (for example, it is possible to slice AnalogSignals and SpikeTrains according to time as well as according to index).

Other Python packages for working with time series data do not fill the niche that Neo does. The core function of Neo is data representation. The Pandas package (http://pandas.pydata.org) is a general toolkit for working with heterogeneous data. Neo contains functionality specific for neural data analysis, such as grouping of channels by anatomical location. Another package, nitime (http://nipy.org/nitime), provides algorithms for time-series analysis that are tailored toward neuroimaging data. In contrast, the Neo package intentionally does not provide algorithms for data analysis since this will vary widely across users. Overall, Neo provides functionality that is specific to neuroscience data (unlike Pandas) but not specific to particular applications within neuroscience (unlike nitime). This means that Neo is ideally situated to be a common format for neuroscientists, who may then analyze the data using the tools provided by other packages as desired.

Because the primary function of Neo is neural data representation, the methods provided fall into two categories: (1) reading from the various formats used by hardware manufacturers; (2) linking the resulting objects in a neuroscientifically meaningful way. The first type of linkage is spatial: AnalogSignal objects may be added to RecordingChannel objects, and RecordingChannel objects added to spatially defined RecordingChannelGroup objects. The second type of linkage is temporal: AnalogSignal objects also belong to a Segment object (representing a period of time), and Segment belongs to Block (representing an entire experiment). Typically the provided IO methods construct the linkages in this hierarchy automatically. The user may then iterate over the data in whichever way is most intuitive for the application: either temporally (trial by trial within a session) or spatially (channel by channel within the brain).

The Neo package is available from the Python Package Index (https://pypi.python.org/pypi/neo/) and as Debian and Ubuntu packages through NeuroDebian (Halchenko and Hanke, 2012). The core of Neo works with both Python 2 (version 2.6 or later) and Python 3 (version 3.2 or later). For some file formats, only Python 2 is currently supported. At the time of writing, the latest version is 0.3.1. Full documentation is available online at http://neuralensemble.org/neo/. There is an extensive test suite, and the project makes use of continuous integration (https://travis-ci.org/NeuralEnsemble/python-neo) to rapidly catch regressions.

## 4. Reading and writing data with multiple file formats

In addition to implementing the Neo object model as a Python API, the Neo Python package also provides a set of input/output (IO) modules for various neurophysiology file formats, including proprietary formats (e.g., Plexon, Spike2, NeuroExplorer, Axon, AlphaOmega, Micromed and Tucker-Davis), the formats used by various open-source and/or freely distributed tools (e.g., Klustakwik, Elan, WinEdr) and generic file formats such as MATLAB, ASCII and HDF5. In most cases, the proprietary formats are read-only, while the more open and generic formats support both reading and writing. The Neo documentation provides guidelines for anyone wishing to add support for a new format.

The inclusion of support for multiple file formats in the Neo package has several benefits: (i) it demonstrates the universality of the object model, which is able to represent the data from all of these different formats; (ii) it is an important part of realizing our goal of improving interoperability of different tools and the ease of sharing data between different projects; (iii) it provides an immediate benefit to users, thus driving uptake of the Neo software; (iv) tool developers can use Neo as a basis and immediately gain the benefit of supporting multiple file formats, without them having to implement such support themselves.

For each file format there is a separate Python class, each of which implements the same interface. Reading data for a given format can be as simple as:


**from** neo.io **import** MyFormatIO
reader = MyFormatIO(filename=“myfile.dat“)
data = reader.read()


However, given the large size of many neurophysiology datasets, it is not always desirable to load all the data into memory at once. The full interface therefore offers more fine-grained control. All read functions have two optional parameters: cascading and lazy. When cascading is set to false, only a single object is loaded and none of its references to other objects are populated. The lazy parameter does not influence whether linked objects are loaded, but determines the treatment of data objects: when lazy is true, the numerical data is not loaded, instead empty data objects are created including all properties and metadata. This allows examining the contents of files with a substantially smaller memory footprint. IO classes can implement a method to load the full version of a lazily loaded object once the numerical data is required.

The IO API also takes into account that a dataset does not necessarily come from a single file. Some software provides recordings in a directory or in a database. Such cases are taken into account through the attribute mode in each IO class. The behavior is changed only at instantiation:


**from** neo.io **import** MyFormat1IO, MyFormat2IO
reader1 = MyFormat1IO(filename=“myfile.dat”)
               # *MyFormat1IO.mode = 'file'*
reader2 = MyFormat2IO(dirname=“/path_to_dataset”)
               # *MyFormat2IO.mode = 'dir'*


Some file formats internally store arrays in a compact continuous way. In such cases, the class implementations use the NumPy “memmap” mechanism. This reduces the memory footprint and allows transparent access to files that do not fit into memory.

For the implementation of file readers, an early difficulty was collecting information about the specifications for proprietary file formats. This tedious task was accomplished using several approaches: (i) asking manufacturers to open the specifications; (ii) reading code from other packages; (iii) reading C header files; (iv) basic reverse engineering with hexadecimal editors. Nowadays, many manufacturers provide their file specifications in the user documentation. The main difficulty remains in the versioning of these specifications. In general, the internal file formats do not change much over time, but sometimes some problematic changes occur, making the classes non backward compatible. This has been taken into account for some file formats. The AxonIO implementation is a good example where two very different versions of the same file format can be read transparently. Unfortunately, this is not the case for all classes and the effort must continue.

## 5. Usage examples

### 5.1. Recording multiple trials from multiple channels

In this example we suppose that we have recorded from an 8-channel probe, and that we have recorded three trials. We therefore have a total of 8 × 3 = 24 signals, each represented by an AnalogSignal object.

**Figure 3:**
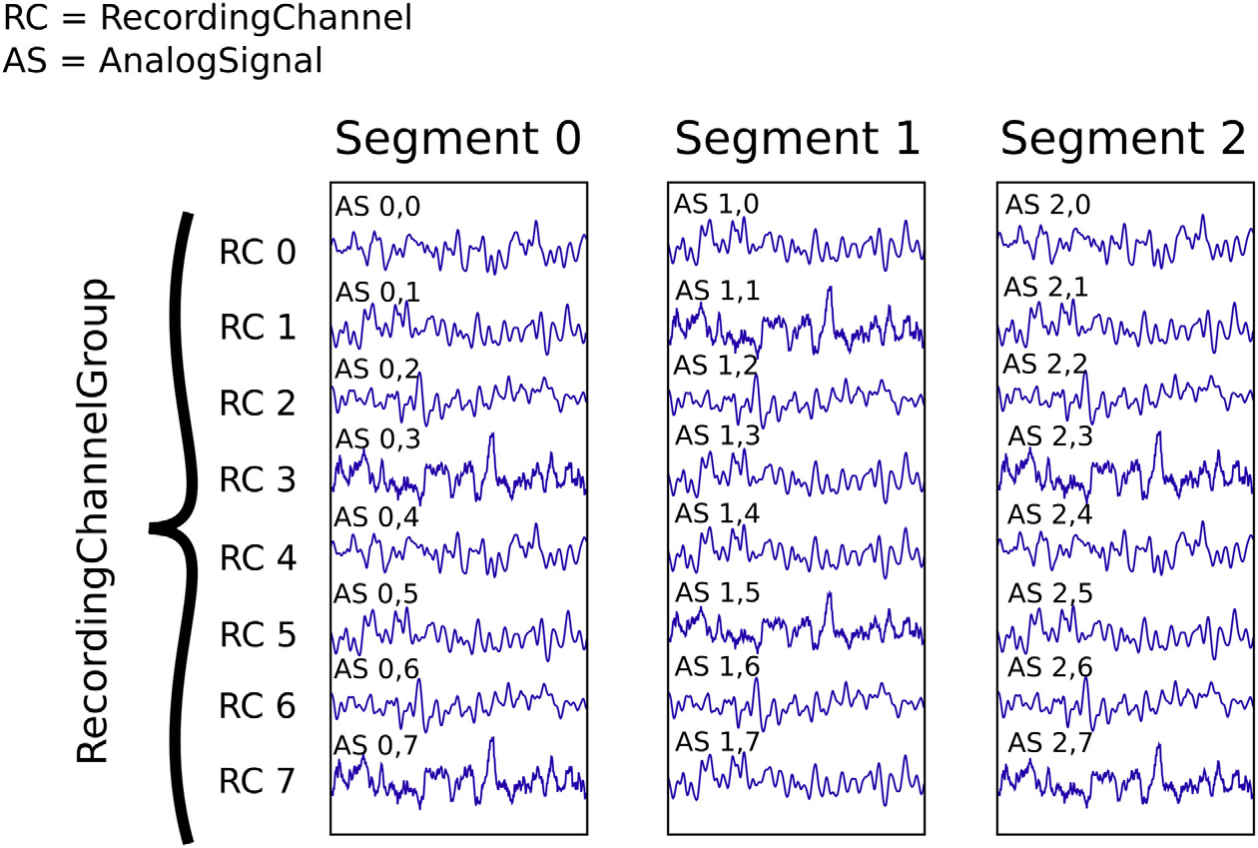
**An illustration of recording multiple trials from multiple channels**. Each Segment contains the data recorded from a single trial. The RecordingChannel objects identify the channel on which a given signal was recorded, and hence link AnalogSignal objects across trials. The RecordingChannelGroup indicates that all channels are recorded from the same 8-channel probe. All the objects shown here are contained in a single Block, which is not explicitly shown.

As shown in Figure 3, our dataset (contained in a single Block, not explicitly shown) contains:

- Three Segment objects, each representing data from a single trial,
- One RecordingChannelGroup, composed of eight RecordingChannel objects.

This information can be traversed or averaged in two different ways. The first is temporal traversal (by segment). For example, suppose you wish to correlate the overall neural response with the stimulus that was delivered in each segment. In this example, we treat each segment in turn, averaging over the channels in each:


**import** numpy as np
**from** matplotlib **import** pyplot as plt

# *We assume that the variable “block” has been*
# *previously loaded from file*

**for** seg **in** block.segments:
    **print**(“Analysing segment %d” % seg.index)

    siglist = seg.analogsignals
    time_points = siglist[0].times
    avg = np.mean(siglist, axis=0)
    # *Average over signals of Segment*

    plt.figure()
    plt.plot(time_points, avg)
    plt.title(“Peak response in segment %d: %f”
              % (seg.index, avg.max()))


The second alternative is spatial traversal of the data (by channel), with averaging over trials. For example, perhaps you wish to see which physical location produces the strongest response, and each stimulus was the same:


# *We assume that our block has only 1*
# RecordingChannelGroup and each *RecordingChannel*
# *only has 1 AnalogSignal*.
rcg = block.recordingchannelgroups[0]
**for** rc **in** rcg.recordingchannels:
    **print**(“Analysing channel %d: %s”
          % (rc.index, rc.name))

    siglist = rc.analogsignals
    time_points = siglist[0].times
    avg = np.mean(siglist, axis=0)
    # *Average over signals of Recording Channel*

    plt.figure()
    plt.plot(time_points, avg)
    plt.title(“Average response on channel %d”
              % rc.index)


### 5.2. Recording spikes from multiple tetrodes

**Figure 4:**
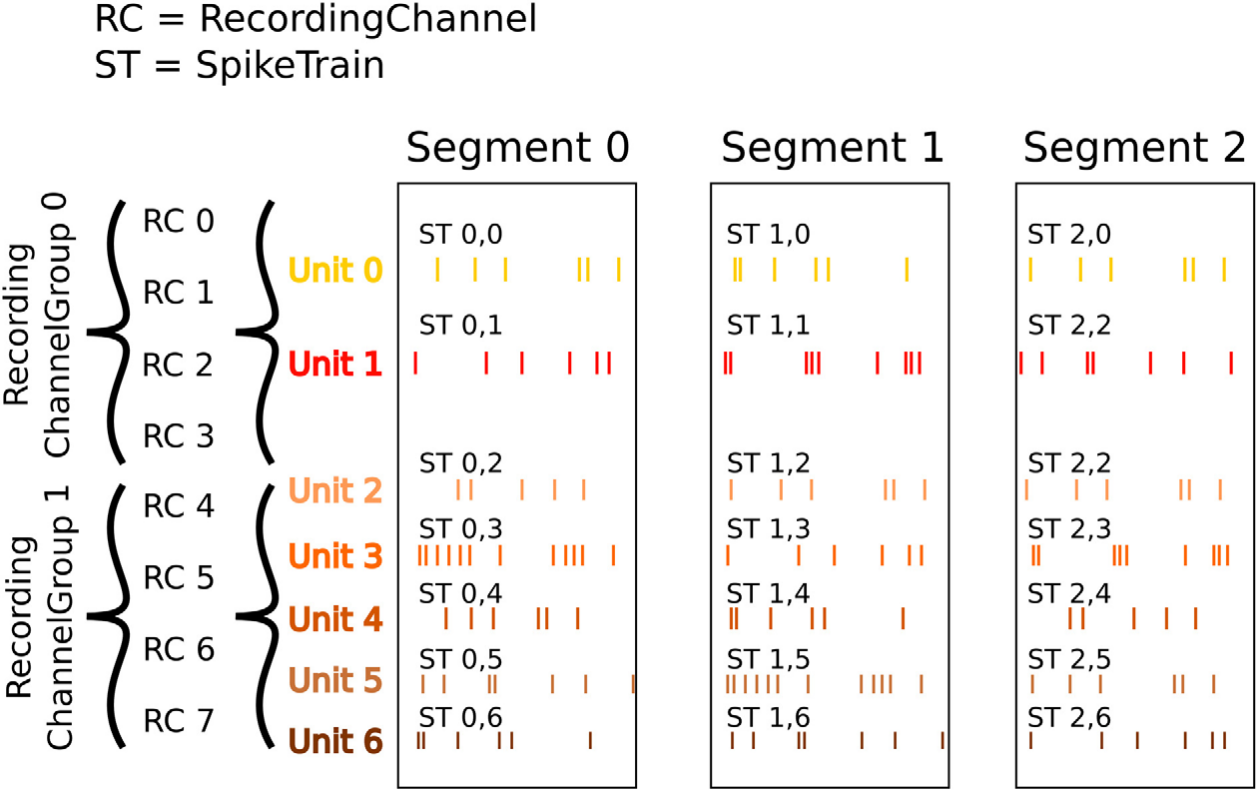
**An illustration of recording spikes from multiple tetrodes**. After spike sorting, two units have been isolated from the first tetrode, and five units from the second. The SpikeTrain objects may optionally also contain the action potential waveforms for each spike (not shown).

Here is a similar example in which we have recorded with two tetrodes and extracted spikes from the extra-cellular signals. The spike times are contained in SpikeTrain objects. As shown in Figure 4, our data set (again contained in a Block) contains:

- Three Segments (one per trial).
- Two RecordingChannelGroups (one per tetrode), which contain:
  - Four RecordingChannels each
  - Two Unit objects (= two neurons) for the first RecordingChannelGroup
  - Five Units for the second RecordingChannelGroup.

In total we have 3 × 7 = 21 SpikeTrains in this Block.

There are three ways to access the spike train data: by segment, by recording channel or by unit.

***5.2.0.1. By segment***. In this example, each Segment represents data from one trial, and we want a peristimulus time histogram (PSTH) for each trial from all units combined:


**for** seg **in** block.segments:
    **print**(“Analysing segment %d” % seg.index)
    stlist = [st - st.t_start
              **for** st **in** seg. spiketrains]
    count, bins = np.histogram(np.hstack(stlist))
    plt.figure()
    plt.bar(bins[:-1], count,
            width=bins[1] - bins[0])
    plt.title(“PSTH in segment %d” % seg.index)


***5.2.0.2. By unit***. Now we can calculate the PSTH averaged over trials for each unit:


**for** unit **in** block.list_units:
    stlist = [st - st.t_start
              **for** st **in** unit. spiketrains]
    count, bins = np.histogram(np.hstack(stlist))
    plt.figure()
    plt.bar(bins[:-1], count,
            width=bins[1] - bins[0])
    plt.title(“PSTH of unit %s” % unit.name)


***5.2.0.3. By recordingchannelgroup***. Here we calculate a PSTH averaged over trials by channel location, blending all units:


**for** rcg **in** block.recordingchannelgroups:
    stlist = []
    **for** unit **in** rcg.units:
        stlist.extend([st - st.t_start
                       **for** st **in** unit. spiketrains])
    count, bins = np.histogram(np.hstack(stlist))
    plt.figure()
    plt.bar(bins[:-1], count,
            width=bins[1] - bins[0])
    plt.title(“PSTH blend of recording channel group
              %s” % rcg.name)


The code samples shown here are extracted from a complete example script available in the Neo source code distribution, see https://github.com/NeuralEnsemble/python-neo/blob/master/examples/generated_data.py.

## 6. Discussion

We have presented here an object-oriented API for handling electrophysiology data in neuroscience, and an implementation of this API in the Python programming language. We have shown how this model is designed to allow for convenient and efficient representation of all kinds of electrophysiological data, from spikes to broadband continuous signals, with support for all common recording technologies, including multi-channel systems like tetrodes. Furthermore, we have illustrated the practical use of the Neo Python package through examples in typical real-world situations.

The generality of the Neo object model for the representation of electrophysiological data coupled with the availability of a large set of IO modules for common file formats in the Python package makes Neo a very convenient and powerful base package to build upon when developing software to manipulate, analyze or display electrophysiological data in Python. It thus fulfils our initial goal as a tool to facilitate data sharing and conversion, as well as software interoperability, with the hope that Neo will become the standard base package for the community of Python developers working with electrophysiological data.

### 6.1. Projects using Neo

The success of the Neo project and the suitability of the object model will be determined by whether other software tools for working with neurophysiology data adopt the Neo object model and whether they gain benefits from doing so. A number of projects and laboratories are already using Neo.

The German INCF Node (G-Node) has adopted the Neo model for the representation of electrophysiological data in its data management platform (https://portal.g-node.org/data/). The purpose of the platform is to provide a centralised system for organising and sharing of electrophysiology data. A major obstacle to data sharing and re-use in the field of electrophysiology is the large variety of data formats. The G-Node platform provides a unified way of representing electrophysiological data according to the Neo object model, as well as automated format conversion utilizing the Neo IO modules. Being compliant with the Neo model, the system's API achieves interoperability with other tools that use the Neo objects and facilitates integration of data access and data analysis. G-Node offers a Python client tool that provides a front-end to the data hosted on the G-Node data platform in terms of the Neo Python objects (Sobolev et al., 2014). The G-Node platform complements the Neo data representation with metadata storage using an unrestrictive format (Grewe et al., 2011), enabling extensive data annotation in a standardized way.

PyNN (Davison et al., 2009) is a Python API for simulator-independent neuronal network simulations. One aspect of simulator independence is reformatting the data produced by each simulator into a common format. Originally, this common format was based on NumPy arrays together with a small amount of metadata, which could be saved to file in a small number of non-standard formats. As of version 0.8, PyNN has adopted Neo as the common format for output data. This has had several benefits: (i) the hierarchical structure of Neo allows much more of the structure of the simulation experiment to be preserved; (ii) a much more complete set of metadata can easily be provided; (iii) a much wider range of file formats is now available; (iv) data recorded in biological experiments and data generated by simulations can now be analyzed and visualized with the same tools; (v) reduced development effort for the PyNN development community, since a large piece of functionality has been “out-sourced” to Neo.

Spyke Viewer (Pröpper and Obermayer, 2013) is a flexible and extensible graphical application for navigating, analysing and visualising electrophysiology data. The central features of Spyke Viewer are the navigation view, filter system, and plug-in architecture. Filters define data subsets of interest. Plug-ins implement data analysis algorithms or visualizations, and Spyke Viewer comes with a variety of plug-ins implementing common neuroscientific plots such as raster plots or correlograms. Spyke Viewer uses Neo as its object model as well as for loading and exporting data. The navigation view allows users to select Neo grouping and container objects, offering a common structure for the data independent of its source format. Filters use properties and annotations of Neo objects to define which objects are shown in the navigation view. Plug-ins operate on Neo data objects. Because of the standard data model provided by Neo, plug-ins work with data from many different formats. This enables Spyke Viewer users to conveniently share methods implemented in plug-ins.

OpenElectrophy (Garcia and Fourcaud-Trocmé, 2009) is a graphical user interface built on top of Neo. This software implements four independent components: (i) SQL database management for Neo objects; (ii) a fast viewer for Neo objects; (iii) a complete off-line spike sorting tool-chain; (iv) a time-frequency toolbox (fast multi-channel wavelet scalogram plotting and transient oscillation detection). The main difficulty when designing a spike sorting tool-chain is the management of data in various and complex situations: (i) the hardware setup may use a single electrode, several independent electrodes, several independent N-trodes or electrode arrays; (ii) the protocol may involve one continuous recording or multiple chunks of recording; (iii) the nature of the recording may be full band signals, filtered signals or pre-detected spikes. Many spike sorting tools take into account only one combination of these situations because it is rather complex to support all the possible combinations. The Neo object model allows all these situations to be dealt with transparently in OpenElectrophy.

Datajongleur (https://github.com/G-Node/Datajongleur) is a collection of scientific data objects. It supports management of data objects with numerical behavior in combination with relational databases, like SQLite (http://www.sqlite.org/, file based), or PostgreSQL (http://www.postgresql.org/, server based). Datajongleur provides a Neo implementation that allows storing Neo objects in a relational database.

As a consequence of the common use of the Neo Python package, it is now straightforward to take recorded data from experiments or simulation data generated by PyNN, store them on the G-Node data platform or in a relational database using Datajongleur, then analyze and visualize them in Spyke Viewer or OpenElectrophy, in a seamless pipeline. Although we have focused in this section on the advantages of Neo for tool developers, the Neo Python package is equally suitable for use by individual scientists analysing their data with their own scripts.

### 6.2. Flexibility and extensibility of the object model

The Neo object model has been designed to be as general as possible while still retaining the ability to clearly represent data objects and express the relationships between them in a way that is natural for neuroscientists. Nevertheless, many experiments in neuroscience combine electrophysiology recordings with recording of other types of data, such as temperature and position in space, and with sensory stimuli. Many such data types are straightforward to represent using the Neo object model. For example, temperature, the angle of a trackball, or an auditory stimulus can all naturally be stored in an AnalogSignal, while a SpikeTrain could be adapted to represent transients (time of occurrence plus waveform) in calcium imaging data.

For data that don't naturally fit into the current objects, it is possible for users to define their own classes that interact with the Neo objects. For example, with calcium imaging data, a user could define their own class to represent the image series, then use AnalogSignal to represent the time course of activity within a region of interest. To store such mixed data, either two separate files can be used, or, with the HDF5 format, both data types can be stored in a single file, using the NeoHdf5IO class to automatically store the time-series data and using an HDF5 library directly to store the imaging data.

### 6.3. Related interoperability efforts

As outlined in the Introduction, there have been a number of previous efforts to solve the problem of incompatible file formats in neurophysiology and related fields, such as clinical electrophysiology.

BioSig (http://biosig.sourceforge.net/) is a cross-platform open source library for biomedical signal processing, available for MATLAB/Octave and for C/C++. Python bindings can be generated from the C/C++ library using SWIG. It includes support for a large number of file formats. Neuroshare (http://neuroshare.sourceforge.net/) is an API that must be implemented as a library by each equipment manufacturer that wishes to support it. The libraries are not in general open source and most of them are only available for Windows. NiBabel (http://nipy.sourceforge.net/nibabel/) is a Python library for accessing common neuroimaging and medical file formats – its goals are very similar to those of Neo, but in a different domain. In the biomedical domain there are a large number of file formats for storage of biomedical time series data (ECG, EEG, etc.; reviewed in Schlögl, 2010), some of which are international standards and others quasi-standards due to widespread uptake. An example of the latter is the European Data Format (EDF and EDF+) which was supported by about 50 companies as of 2004 (Kemp, 2004). The GDF format (Schlögl, 2006) is a more recent development that addresses many of the limitations of earlier formats. SignalML (Durka and Ircha, 2004) is an alternative approach, defining a markup language for describing file formats so as to allow automatic conversion. Kemp (2004) compares EDF+ and SignalML, concluding that the SignalML approach is more flexible but that this flexibility comes at a price in terms of stability and increased effort for users.

We expect that Neo will be interoperable with, and broadly complementary to these existing efforts. For example, the Neo Python package already offers a NeuroshareIO module for reading data files via the Neuroshare libraries, and it should be straightforward to add IO modules for EDF/EDF+, GDF and the HDF5-based standard file format being developed by the INCF. The particular advantages of the Neo Python package are (i) its rich object model can capture all of the complexity typically found in neurophysiology experimental protocols; (ii) it is written in pure Python without any C/C++ dependencies except the widely available NumPy package; as such it is easy to install and highly cross-platform; (iii) it focuses on neuroscience research, whereas most of the other tools cited above have a biomedical focus; (iv) by using the Python programming language, users of Neo gain all of the benefits associated with that language, such as readability, increased productivity, ease of use for system integration, and access to a powerful ecosystem for scientific computing.

### 6.4. Sustainability

Neo is developed as an open, collaborative project at http://neuralensemble.org/neo, using GitHub for version control and issue tracking. Anyone is welcome to propose and implement changes, which will then be reviewed by one or more of the other contributors, and a decision on whether to accept the changes taken by consensus. 19 people from ten separate institutions have at some time contributed code or documentation to the Neo project; six people from five institutions have contributed within the past 12 months. This distributed development effort, in which the collaboration arises informally from shared needs and interests rather than formally through grant funding, gives us confidence that Neo development is sustainable.

### 6.5. Future plans

The Neo object model was designed after discussion between a group of scientists and engineers, both developers and potential users, from several laboratories. We consider the essential elements of the object model to be stable. However, as users and developers gain experience with the software, some evolution is to be expected. It will be necessary to find a compromise between changes to enhance usability and support new use cases, and the stability needed for a low-level library on top of which other software is built.

Among the improvements already envisaged are the following: (i) some simplification of the object model, in particular merging of the scalar and array representations (e.g., AnalogSignal and AnalogsignalArray); (ii) addition of an image sequence object, to support calcium- and voltage-sensitive-dye imaging experiments, which are closely related to electrophysiology.

We also plan a number of improvements to the Python implementation: (i) improved memory management for IO. Despite the lazy/cascade mechanism, most Neo IO classes do not allow loading only a chunk or a subset of data; (ii) efficiency improvements. Some relationships are not efficient for querying (filtering) an object when the dataset is large. For instance, linking a SpikeTrain with its associated AnalogSignal is not currently straightforward.

The object model is intended to be implementable in other languages besides Python. We have concentrated our initial efforts on one language in order to build up a critical mass of code to support the object model. An important future direction will be reaching out to users of other languages, such as MATLAB, and providing support for the object model. To this end, we have created documentation for using Neo-structured data in MATLAB at http://pythonhosted.org/neo/io.html#neo.io.NeoMatlabIO.

The development of the Neo object model has so far focused on the representation of recorded data, as typically stored in data files during an electrophysiology experiment. For broader use it would be desirable to also support data resulting from the processing and analysis of such data. Ideally, the object model should enable representing the relationship between analysis results and the original data. Instead of leaving the definition of corresponding objects to each user, it would make sense to extend the Neo model by a minimal set of definitions for generic scientific data along with provenance information. Such developments should be coordinated with international standardization efforts, such as those of the INCF Data Sharing Program (Teeters et al., 2013).

### Conflict of interest statement

The authors declare that the research was conducted in the absence of any commercial or financial relationships that could be construed as a potential conflict of interest.
